# Program of rehabilitative exercise and education to avert vascular events after non-disabling stroke or transient ischemic attack (PREVENT Trial): a multi-centred, randomised controlled trial

**DOI:** 10.1186/1471-2377-10-122

**Published:** 2010-12-08

**Authors:** Marilyn MacKay-Lyons, Gordon Gubitz, Nicholas Giacomantonio, Howard Wightman, David Marsters, Kara Thompson, Chris Blanchard, Gail Eskes, Marianne Thornton

**Affiliations:** 1 School of Physiotherapy, Dalhousie University, Halifax, Nova Scotia, Canada; 2 Department of Neurology, QEII Health Sciences Centre, Halifax, Nova Scotia, Canada; 3 Department of Cardiology, QEII Health Sciences Centre, Halifax, Nova Scotia, Canada; 4 Department of Medicine, Valley Regional Hospital, Kentville, Nova Scotia, Canada; 5 Department of Medicine, QEII Health Sciences Centre, Halifax, Nova Scotia, Canada; 6 Department of Psychiatry, QEII Health Sciences Centre, Halifax, Nova Scotia, Canada

## Abstract

**Background:**

Despite lack of outward signs, most individuals after non-disabling stroke (NDS) and transient ischemic attack (TIA) have significant cardiovascular and cerebrovascular disease and are at high risk of a major stroke, hospitalization for other vascular events, or death. Most have multiple modifiable risk factors (e.g., hypertension, physical inactivity, hyperlipidaemia, diabetes, tobacco consumption, psychological stress). In addition, accelerated rates of depression, cognitive decline, and poor quality of sleep have been reported following TIA, which correlate with poor functional outcomes and reduced quality of life. Thus, NSD and TIA are important warning signs that should not be overlooked. The challenge is not unlike that facing other 'silent' conditions - to identify a model of care that is effective in changing people's current behaviors in order to avert further morbidity.

**Methods/Design:**

A single blind, randomized controlled trial will be conducted at two sites to compare the effectiveness of a program of rehabilitative exercise and education versus usual care in modifying vascular risk factors in adults after NDS/TIA. 250 adults within 90 days of being diagnosed with NDS/TIA will be randomly allocated to a 12-week program of exercise and education (PREVENT) or to an outpatient clinic assessment and discussion of secondary prevention recommendations with return clinic visits as indicated (USUAL CARE). Primary outcome measures will include blood pressure, waist circumference, 12-hour fasting lipid profile, and 12-hour fasting glucose/hemoglobin A1c. Secondary measures will include exercise capacity, walking endurance, physical activity, cognitive function, depression, goal attainment and health-related quality of life. Outcome assessment will be conducted at baseline, post-intervention, and 6- and 12-month follow-ups. Direct health care costs incurred over one year by PREVENT versus USUAL CARE participants will also be compared. Ethical approval for the trial has been obtained from the relevant Human Research Ethics Boards.

**Discussion:**

Whether timely delivery of an adapted cardiac rehabilitation model is effective in attaining and maintaining vascular risk reduction targets in adults after NDS/TIA is not yet known. We anticipate that the findings of this trial will make a meaningful contribution to the knowledge base regarding secondary stroke prevention.

**Trial registration:**

This trial is registered with the Clinical Trials.gov Registry (NCT00885456).

## Background

"Little stroke, big trouble", the theme of World Stroke Day 2008, speaks to the importance of heeding non-disabling stroke (NDS) and transient ischemic attack (TIA) as critical warning signs of further, more debilitating vascular events or death [1]. (Since the etiology and prognosis of NDS and TIA are essentially the same [2], hereafter they will be referred to as a single entity - NDS/TIA.) Until recently, the prognosis of NDS/TIA was deemed relatively benign because NDS/TIA appears to leave minimal impairment and no overt long-term effects [3]. However, many survivors of NDS/TIA have significant cardiovascular and cerebrovascular disease and a high probability of secondary stroke or death [4] - from 10% in the low-risk group, to 19% in moderate-risk group and 31% in high-risk group [5]. Thus, secondary prevention strategies are needed to avoid disabling stroke, the fourth leading cause of disease burden and the second leading cause of death among adults worldwide [6]. Moreover, recurrent strokes contribute disproportionately to the overall personal and economic burden associated with stroke [7] because of higher rates of institutionalisation and fatality than primary strokes [8].

Individuals presenting with NDS/TIA typically have significant atherosclerotic lesions throughout their vascular system and often manifest co-morbid cardiovascular disease (CVD) [9,10]. Not surprisingly, cardiac disease is the leading cause of death in stroke survivors [11,12]. It follows that CVD and stroke share common risk factors. Among the modifiable factors are hypertension, diabetes, dyslipidemia, physical inactivity, obesity, excessive alcohol consumption, and cigarette smoking [13]. Systolic blood pressure (SBP) is the most robust independent predictor of secondary vascular events such as myocardial infarction or recurrent stroke [14]. Diabetes is a strong independent predictor of stroke during the first year post-TIA, with rate of stroke recurrence being substantially higher in the diabetic stroke population [15]. Although dyslipidemia is less well established as a risk factor for stroke than for cardiac disease [16], recent guidelines for patients post-TIA include the administration of statin therapy with intensive lipid-lowering effects [17].

Both physical inactivity [18] and abdominal obesity [19,20] are independent risk factors for primary stroke. We documented that mean exercise capacity (peak oxygen consumption, VO_2_peak) at one-month post-stroke was only 60% of normative values, increasing to 71% at six months post-stroke [21]. Cigarette smoking has a causal relationship to atheroscelerosis, which explains why smoking is a major independent risk factor for stroke [22]. Heavy alcohol use (> 5 drinks/day) is a factor in stroke recurrence [23]. Metabolic syndrome, or clustering of vascular risk factors, is defined by the International Diabetes Federation as central obesity plus any two of the following: hypertension, hypertriglyceremia, low high-density lipoprotein-cholesterol (HDL-C), and insulin resistance [24]. This syndrome is critical in the development of recurrent ischemic stroke [25] and is also associated with silent brain infarction independent of other risk factors [26]. Control of the risk factors associated with metabolic syndrome is inadequate for people post-TIA or stroke [27] In a cohort of stroke survivors, 67% were overweight or obese, 80% had pre-hypertension or hypertension, 34% had low HDL and 45% had impaired fasting glucose [28]. Among patients post-stroke entering exercise studies, 99% of participants had at least one uncontrolled risk factor and 91% had two or more inadequately treated risk factors [28], figures comparable to what we reported in participants beginning stroke rehabilitation [29]. The problem is compounded by an acute lack of awareness and knowledge of cardiovascular risk factors among the NDS/TIA population [30]. Collectively, these findings suggest that there is considerable room for improvement in vascular risk factor management following NDS/TIA.

For people with CVD or diabetes, cardiac rehabilitation programs, consisting of exercise, education and lifestyle change counselling, improve all of the modifiable risk factors discussed above. Two large trials reported favourable effects of multi-modal cardiac rehabilitation on hypertension [31,32]. Cochrane reviews concluded that exercise alone [33], or the combination of exercise and dietary advice [34], also improve glycemic control (decrease in hemoglobin A1c) in people with type 2 diabetes. Benefits of group-based exercise sessions people with type 2 diabetes include reductions in SBP, hemoglobin A1c, fasting blood glucose levels, body weight and need for diabetes medication, as well as enhanced diabetes knowledge [35]. A ~50% reduction in risk of vascular events over 7.8 year period was reported following an intensive program of exercise, dietary modification and pharmacotherapy for people with type 2 diabetes [36]. Sustained moderate weight loss, achieved through lifestyle interventions (diet and exercise), is effective for the prevention and treatment of hypertension, diabetes and dyslipidemia [37,38]. Dietary advice alone has been shown to improve lipid profiles [34]. A systematic review found that comprehensive cardiac rehabilitation, but not exercise alone, induced reductions in total cholesterol, LDL-C and triglycerides, with no effect on HDL-C [33]. Reviews of multi-modal cardiac rehabilitation programs [33] and distribution of self-help education materials[39] have revealed little effect on smoking cessation.

Burgeoning evidence indicates that aerobic exercise not only reduces mortality in cardiac populations [32,40,41], but benefits mood [42], diminishes anxiety and depression [43], improves cognition [44,45] and decreases risk of dementia in vascular disease [46,47]. These additional benefits are relevant because people with TIA/NDS can have subtle but persistent neuropsychological deficits [48] similar to those accompanying hypertension [49]. As well, reducing depressive symptomatology - a condition found in up to 70% people post-TIA [50] - has been linked to decreased risk of recurrent cerebrovascular events [51].

There is good reason to suggest that risk factor management for NDS/TIA should be aligned with that of cardiovascular conditions, given that secondary risk factors, risk of recurrence, and the potential benefits of treatment are similar [2]. Preliminary support for a multi-faceted approach to secondary stroke prevention comes from a modeling study in which the authors concluded, ″At least four-fifths of recurrent vascular events in patients with cerebrovascular disease might be prevented by application of a comprehensive, multi-factorial approach″ [52]. A non-randomized study of a 3-month exercise program for patients within six months of NDS/TIA demonstrated improvements in walking endurance and exercise capacity [53]. Another non-randomized study of an 8-week educational program for people post-NDS/TIA demonstrated improved knowledge regarding stroke warning signs, medication adherence, blood pressure monitoring and reduction in salt intake [54]. A recent pilot randomized trial of a 10-week cardiac rehabilitation program for patients post-stroke demonstrated greater improvement in cardiac risk scores in the experimental group than the usual care group [55]. However, high-level evidence of risk factor reduction through non-pharmacological approaches is lacking [56]. For the most part, secondary stroke prevention trials have been short-term, focusing on pharmacological interventions (for example [57,58]) and endarterectomy for patient with surgical grade carotid stenosis [59].

Little provision is made in current health services for modification of lifestyle factors such as cardiovascular fitness, weight optimization and healthy eating for long-term secondary prevention in patients with NDS/TIA. This situation is at odds with recommendations supporting a combination of comprehensive lifestyle interventions and pharmacological therapy to prevent recurrent stroke and acute cardiac events in stroke survivors [60,61]. Indeed, Myint and colleagues [62] postulated that behavioral modifications may be as important as anti-hypertensive and cholesterol lowering agents in secondary prevention of stroke.

A major thrust in public health over the next decade will be to focus attention on identification and management of vascular risk factors. Although primary prevention is the ultimate goal, resources will need to be allocated to those who are at high risk and most likely to gain the greatest benefit. One such group is people who have experienced a NDS/TIA. The main objective of the PREVENT trial is to determine whether the timely delivery of a comprehensive program of secondary stroke prevention services and coordinated care can improve long-term vascular risk reduction for patients after NDS or TIA. The primary aim is to compare the effectiveness of a program of rehabilitative exercise and education (PREVENT) versus usual care (USUAL CARE) in reducing vascular risk factors in people who have had a NDS or TIA. Secondary aims are to compare between PREVENT and USUAL CARE groups (i) exercise capacity, walking endurance, daily physical activity, number of cigarettes smoked daily, medication adherence; (ii) fatigue, cognitive function, depression, health-related goal attainment and health-related quality of life; (iii) direct health costs incurred over a 1-year period; (iv) reduction in secondary vascular events, within the limitations in sample size and trial duration. We anticipate that, in comparison to USUAL CARE, timely delivery of PREVENT will produce clinically important and sustainable improvements in the outcome variables.

## Methods/design

All procedures involved in this trial with human participants will be conducted in compliance with institutional ethical standards and the Helsinki Declaration. Ethical approval to conduct the trial has been granted by the Capital District Research Ethics Board (protocol CDHA-RS/2009-371) and the Annapolis Valley District Research Ethics Board (protocol #2009-005).

### Study Design

**Figure 1:**
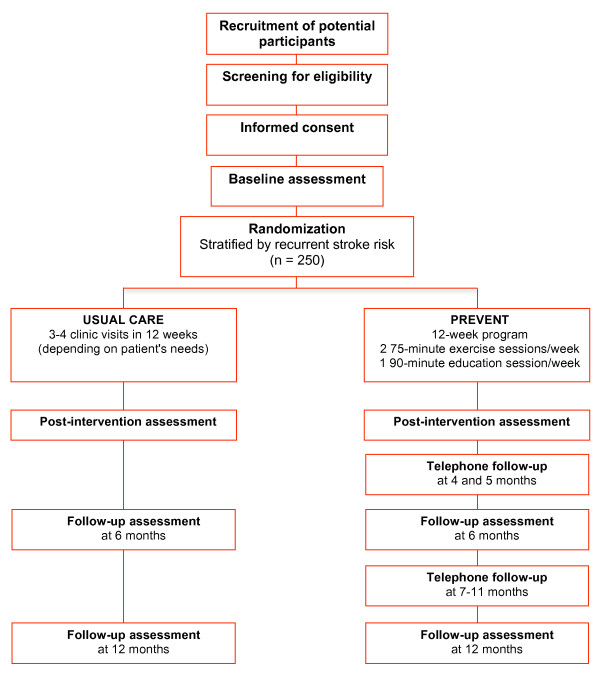
**Flow chart outlining the study protocol**.

This is a two parallel group, four-site, single-blinded, randomized trial with group assignment stratified using the Stroke Prognosis Instrument II (SPI-II) [5]. Figure 1 illustrates the sequencing of the study protocol.

### Setting

Four community-based cardiac rehabilitation programs, three located in one urban/suburban environment (Halifax, Nova Scotia, Canada) and one in a more rural setting (Kentville, Nova Scotia, Canada) have been selected at intervention sites based on recruitment potential, adherence to the national guidelines for hyperacute and acute management of NDS and TIA [60], and availability of existing programs that could be adapted to meet the protocol for this trial.

### Participants

#### Inclusion Criteria

Participants will include males and females, over 17 years of age, who are within 90 days of diagnosis of first probable or definite TIA (symptom resolution in less than 24 hours) or ischemic or hemorrhagic NDS (National Institutes of Health Stroke Scale less than 6) [63] for which medical attention was sought.

#### Exclusion Criteria

Potential participants will be excluded if they present with contraindications to exercise testing and training, in accordance with American College of Sports Medicine Guidelines (ACSM) [64] and/or are participating in another study that could potentially confound the outcomes of this trial.

#### Participant recruitment

Recruitment pamphlets approved by the Research Ethics Board and checklists outlining the inclusion/exclusion criteria will be distributed in emergency rooms, physicians' offices, stroke units, and outpatient stroke clinics. The neurologists or internists directing acute stroke services and emergency room physicians in the trial sites will be trained to identify patients who meet the selection criteria and will notify the PREVENT Trial site coordinator of potential research subjects. The site coordinators will also communicate directly with the recruitment locations twice weekly for potential participants and will provide ongoing education, recruitment flyers, and reminders about the trial. The site coordinators will also review each candidate's health record for inclusion/exclusion criteria and record basic demographic and medical information.

Eligible patients will be approached by the site coordinator, who will briefly explain the purpose of the study and ask if they have an interest in participating. If a potential participant expresses interest, the study will be explained in detail, all questions will be addressed and the participant will be asked to sign the consent form approved by the Research Ethics Board of that site. The family physicians of the participants will be informed of intent to participate. The Environmental Supports for Physical Activity Questionnaire [65] will also be administered. Those patients who do not wish to participate will be asked to complete a brief questionnaire about their daily physical activity, to assess the representativeness of the final study sample.

#### Sample Size

Systolic blood pressure, the most robust predictor of secondary vascular events [14], was selected as the outcome variable for sample size calculation. Using estimates obtained from our pilot study, a sample size of 119 in each group will have 80% power to detect a difference in means of 4.9 mmHg, assuming that the common standard deviation is 13.4 using a two group t-test with a 0.05 two-sided significance level. We have allowed for an overall attrition rate of 5% [55], thus increasing the sample size to 125 per group (250 in total). The trial will not be sufficiently powered to determine the effectiveness of the PREVENT intervention in reducing recurrent TIA, stroke or cardiac events. Thus, it will be exploratory in this regard and will help inform the design of a future, more extensive trial.

#### Randomization Procedures

Participants will be randomized to one of two interventions: USUAL CARE or PREVENT. To ensure comparable groups in terms of neurological status, group assignment will be stratified using the SPI-II, which assesses long-term risk of stroke post-TIA or NDS using a 15-point scale [5]. Two strata will be used: 1. SPI-II Level 1 (0-3 points; low-risk), 2. SPI-II Level 2 (4-7 points; moderate-risk), and Level 3 (8-15 points; high-risk). An individual not associated with the study will use a computerized random number generator to randomly assign subjects to group. Allocation concealment will be ensured by using opaque, a sealed envelope containing group assignment, which will be revealed, in the presence of the participant, after completion of the baseline assessment. The participants (who are necessarily unblinded) will be requested to avoid informing blinded evaluators of their group assignment.

### Outcome Measurement

Given the long duration and multi-modal nature of the interventions, a range of outcome measures will be used to ensure evaluation of relevant variables that span the domains of the International Classification of Functioning [66]. The variables and measurement tools that will be employed are summarized in Table 1. These measures were selected based on the primary and secondary aims of the trial, clinical relevance, psychometric qualities, and feasibility (availability, cost and administration time). Evaluations will be conducted over a 2-day assessment period at baseline, post-intervention, six months, and 12 months by trained assessors blinded to knowledge of study hypotheses and group assignment. At each assessment the assessors will be asked to record their 'guess' as to the group assignment of each participant.

**Table 1:** Outcome variables, measurement tools and assessment schedule

Variable	Measurement tool	Baseline	Post-intervention	6-month follow-up	12-month follow-up
*Primary measures*					

Blood pressure	Sphygmomanometer [67]	√	√	√	√

Waist girth	Tape measure [68]	√	√	√	√

Lipid profile*	Biochemical analysis	√	√	√	√

Fasting serum glucose	Biochemical analysis	√	√	√	√

Haemoglobin A1c	Biochemical analysis	√	√	√	√

*Secondary measures*					

Aerobic fitness	Peak VO_2 _using maximal treadmill stress test (ramp protocol)	√	√		√

Lower extremity function	Short Physical Performance Test [69]	√	√	√	√

Walking endurance	Six-Minute Walk Test [70]	√	√	√	√

Physical activity	Step Watch™accelerometers	√	√		√

	International Physical Activity Questionnaire [71]	√	√	√	√

Fatigue levels	Fatigue Assessment Scale [72]	√	√	√	√

Cognition	Montreal Cognitive Assessment [73]	√			√

Mental health	Hospital Anxiety and Depression Scale [74]	√	√	√	√

Quality of sleep	Pittsburg Sleep Quality Index [75]	√	√	√	√

Tobacco use	Self-report, using a health passport	√	√	√	√

Health care utilization	Self-report, using a health passport	√	√	√	√

Medication adherence	Self-report, using a health passport	√	√	√	√

Health-related quality of life	Medical Outcomes Study Short-form 36-item Questionnaire [76]	√	√		√

Health-related goals	Goal Attainment Scaling [77]	√	√	√	√

Secondary vascular events**	Health record abstraction	√	√	√	√

*Includes total cholesterol, high-density lipoprotein cholesterol (HDL-C), low-density lipoprotein cholesterol (LDL-C), triglycerides

**Includes second stroke, second TIA, acute coronary syndrome (i.e., unstable angina and myocardial infarction), heart failure, and vascular death

### Interventions

#### USUAL CARE Intervention

Following completion of the acute management of TIA and NDS, USUAL CARE participants will be referred to the site's outpatient Neurovascular Clinic, staffed by neurologists/internists and nurses, for a neurological and health assessment, counselling regarding stroke/TIA and diagnostic test results, and assessment, modification and education of secondary prevention factors (i.e., dietary intake, lipid profile, adherence to medication regime, physical activity, smoking, alcohol intake, elf-referral to weight loss and smoking cessation programs) [78]. Required therapeutic interventions will be initiated or adjusted (e.g., medications, diagnostic/laboratory tests) and referrals will be sent to appropriate team members or clinics (e.g. social worker, physiotherapist, occupational therapist, dietician, speech language pathologist, diabetes clinic, hypertension clinic, anticoagulation clinic, smoking cessation program). Follow-up care will be provided by return clinic visits, with an average of three visits in total. Those patients who no longer require follow-up will be referred back to their primary care physician.

#### PREVENT intervention

After completing the acute management of TIA and NDS, PREVENT participants will participate in a multi-modal, case-managed program of exercise and education. Because this intervention is behavior-focused (e.g., encouraging daily physical activity, health eating, smoking cessation, medication use adherence), several strategies known to facilitate and sustain behavioral change have been incorporated into the design [79,80]:

(i) Each participant will meet with the PREVENT physicians and program providers to identify personal goals/barriers/possible solutions related to risk factor targets and lifestyle modifications, based on the baseline assessment and the participant's needs and values.

(ii) Each participant will be provided with a user-friendly health passport in which the participant and PREVENT staff will maintain documentation regarding health-related appointments, assessment results, medications, BP, cigarettes smoked, lipid profile, blood glucose, body weight, waist circumference, daily physical activity, dietary intake, as well as goal attainment related to these domains. All information in the passport will be written in 'plain language' (i.e., Grade 8 reading level).

(iii) Use of positive reinforcement (encouragement, positive feedback).

(iv) Use of adult learning strategies (interactive educational sessions, participant involvement in content selection).

(v) After completion of the formal program, telephone follow-up will be implemented (see below). In addition, individual therapeutic interventions will be initiated or adjusted, as described above for usual care.

##### Group exercise component

75-minute group exercise sessions, lead by a physiotherapist or kinesiologist, will be held twice weekly for 12 weeks. The sessions will involve (i) 5-10 minutes of warm-up exercises, (ii) 15 minutes of progressive resistance training of major extremity muscles, (iii) 30 minutes of aerobic training consisting of three 10-minute stations of treadmill walking, stationary cycling and stepping at an initial intensity of 40-70% of heart rate reserve, as determined by stress test results; (iv) 5-10 minutes of cool-down exercise; (v) progressive resistive strengthening of major upper and lower extremity muscle groups. Heart rate will be continuously monitored, and BP and blood sugars will be measured before and after exercise, as indicated. Both strengthening and aerobic training will be prescribed and progressed according to ACSM guidelines [64].

##### Home exercise component

Group exercise sessions will be supplemented by 30 minutes of home cardiovascular exercise, 3-4 days/week at a rating of perceived exertion (RPE) of 3-5 on the Borg 0-10 scale [81,82].

***Education component ***One 1.5-hour group session will be conducted weekly by a multi-disciplinary team of health professionals. The schedule for the educational sessions and topics of discussion are outlined in Table 2. Family members/caregivers of participants will be encouraged to attend. Powerpoint slides will be used to ensure consistency of information across sites. Participants will also have access to scheduled grocery shopping tours and cooking demonstrations to further support program objectives.

**Table 2:** Schedule and discussion topics for educational sessions.

Week 1	Heart healthy eating: The basics
Week 2	Setting of health-related goals

Week 3	Exercise: The basics

Week 4	Cardiovascular risk factors and BP self-monitoring

Week 5	Nutrition: Building on the basics (including a 3-day food record analysis by the dietician)

Week 6	Exercise: Building on the basics

Week 7	Cardiovascular medications

Week 8	Healthy weight

Week 9	Smoking cessation

Week 10	Stress & coping

Week 11	Fine-tuning healthy eating

Week 12	Wrap-up

##### Intervention audits

All professionals involved in delivering PREVENT will receive standardized training to ensure consistency of program delivery across sites. To appraise the actual level of consistency, audits will be conducted at random intervals by the trial coordinator. Any deviations from the protocol will be rectified by the site PREVENT team.

##### Post-program follow-up

After the 12-week program, PREVENT participants will be provided with a written maintenance program based on their long-term goals. Participants will be requested to maintain their health passport and will be phoned monthly by the site coordinator until the 12-month follow-up as a means of monitoring of use of health passport, health status, health care utilization and adherence to the maintenance program.

### Data management and analysis

Data collected at each site will be recorded on standardized forms and entered into a secure database using a centralized, web-based data entry system that contains quality control checks (e.g., range checks, notification of missing data). An independent Data Safety and Monitoring Board, consisting a cardiologist, neurologist and a statistician, has been set up to review mid-point results (or at any time that a serious adverse event occurs) and inform the investigators on issues of trial safety.

Descriptive statistics will be calculated for dependent and independent variables. Demographic and clinical characteristics of subjects in the experimental and control groups will be compared on admission using 2-sample t-tests (continuous data) and Chi-square analysis (nominal data). Nonparametric methods will be used when assumptions of normality are violated. Similar analyses will be used to compare characteristics of (i) patients who consent to participate versus those who do not; (ii) subjects who complete the study versus those who withdraw, in order to determine the representativeness of the sample. A mixed effects analysis of variance with repeated measures will be used to model the treatment effects for variables that are interval or ratio and conform to the assumptions of normality (i.e., BP, waist girth, total cholesterol, HDL-C, LDL-C, triglycerides, serum glucose, hemoglobin A1c, peak VO_2_, Six-Minute Walk Test, step counts). Non-parametric tests (Mann Whitney and Kruskall Wallis tests) will be used for ordinal level data (i.e., International Physical Activity Questionnaire, Fatigue Assessment Scale, Montreal Cognitive Assessment, Hospital Anxiety and Depression Scale, Pittsburg Sleep Quality Index, Goal Attainment Scaling) or where a normal data distribution is not found. To control for baseline differences between groups, variables that are significantly different at baseline will serve as covariates in the analyses. Data will be analyzed on an intention-to-treat basis. Significant omnibus effects will be probed with t-tests, using the Bonferroni correction to control for multiple comparisons. Multiple regression will be conducted where appropriate to examine relationships among variables. Software will include Access and SAS version 9.1. Alpha level will be < 0.05.

## Discussion

The main focus of PREVENT Trial is to investigate the role of aerobic exercise, education and lifestyle counselling in reducing vascular risk factors of people after TIA or NDS. This trial will contribute much needed evidence to begin answering important clinical questions regarding the role of non-pharmacological interventions, modelled after cardiac rehabilitation programs, in preventing second stroke in the often under-served patient populations involved in this trial. Although the trial is not sufficiently powered to determine the effectiveness of PREVENT on hard clinical outcomes such as second vascular events or death, we will compare occurrence rates between groups in order to inform future, larger scale trials.

This multi-site RCT will also explore the impact of the PREVENT program on cardiovascular fitness, walking tolerance, fatigue, cognition, mental health, quality of sleep, medication adherence, tobacco use, and health-related quality of life, which may be mitigating factors in reducing vascular risk factors, and, ultimately, stroke recurrence. As well, comparison of the direct health costs of the PREVENT and USUAL CARE groups over the course of the study will provide important preliminary information related to cost effectiveness and sustainability of multi-modal, behavioral interventions such as PREVENT.

## Current study status

Enrolling Patients

## Competing interests

The authors declare that they have no competing interests.

## Authors' contributions

MM-L: led the conceptualization, design, development, funding applications and implementation of this research protocol. MM-L was the primary author for this manuscript. GG: co-led the conceptualization, design, development, and implementation of this research protocol and contributed to the writing of this manuscript. NG, HW, DM, GE: contributed to the design, development, and implementation of this research protocol and the writing of this manuscript. KT: led the development of the data management protocol and statistical analysis plan and contributed to the writing of this manuscript. CB: contributed to the conceptualization, design, and development of this research protocol and the writing of this manuscript. MT: assisted in the design of this protocol.

All authors read and approved of the manuscript.

## Pre-publication history

The pre-publication history for this paper can be accessed here:

http://www.biomedcentral.com/1471-2377/10/122/prepub
